# Cardiorespiratory Dynamic Response to Mental Stress: A Multivariate Time-Frequency Analysis

**DOI:** 10.1155/2013/451857

**Published:** 2013-12-10

**Authors:** Devy Widjaja, Michele Orini, Elke Vlemincx, Sabine Van Huffel

**Affiliations:** ^1^KU Leuven, Department of Electrical Engineering (ESAT), STADIUS, Kasteelpark Arenberg 10, P.O. Box 2446, 3001 Leuven, Belgium; ^2^iMinds, Future Health Department, Kasteelpark Arenberg 10, P.O. Box 2446, 3001 Leuven, Belgium; ^3^University College of London, Institute of Cardiovascular Science, 66 Gower Street, London WC1E 6BT, UK; ^4^KU Leuven, Department of Psychology and Educational Sciences, Tiensestraat 102, 3000 Leuven, Belgium

## Abstract

Mental stress is a growing problem in our society. In order to deal with this, it is important to understand the underlying stress mechanisms. In this study, we aim to determine how the cardiorespiratory interactions are affected by mental arithmetic stress and attention. We conduct cross time-frequency (TF) analyses to assess the cardiorespiratory coupling. In addition, we introduce partial TF spectra to separate variations in the RR interval series that are linearly related to respiration from RR interval variations (RRV) that are not related to respiration. The performance of partial spectra is evaluated in two simulation studies. Time-varying parameters, such as instantaneous powers and frequencies, are derived from the computed spectra. Statistical analysis is carried out continuously in time to evaluate the dynamic response to mental stress and attention. The results show an increased heart and respiratory rate during stress and attention, compared to a resting condition. Also a fast reduction in vagal activity is noted. The partial TF analysis reveals a faster reduction of RRV power related to (3 s) than unrelated to (30 s) respiration, demonstrating that the autonomic response to mental stress is driven by mechanisms characterized by different temporal scales.

## 1. Introduction

Stress research has gained growing interest in the last decades. Results from the European Working Conditions Survey in 2000 suggested that 28% of all work-related health problems can be attributed to stress. Work absence associated with stress not only brings along highcosts; for example, in The Netherlands, they estimate a cost of 3 billion € per year [1], but it is also linked with serious health problems such as cardiovascular diseases [2, 3] and musculoskeletal disorders [1, 4, 5]. It is therefore important to identify the mechanisms underlying stress, such as physiological mechanisms.

The autonomic nervous system (ANS) dynamically coordinates, among others, cardiovascular variables (such as heart rate and contractility, blood pressure, and peripheral resistance), respiration, and complex interactions between them. The impact of stress on the cardiovascular system has been investigated extensively via the variability of the RR interval variation (RRV) series [6–10]. RRV analysis is widely used to assess the functioning of the ANS. Starting from the RR interval series, several RRV indices that quantify the activity of the ANS have been proposed [11]. In the power spectrum of RRV, a low-frequency (LF) band (0.04–0.15 Hz) and a high-frequency (HF) band (0.15–0.40 Hz) are defined. LF power is linked to both sympathetic and vagal activity, while HF power is only coupled to vagal outflow. All studies reported reduced vagal modulation and RRV and an increased sympathovagal balance during mental stress.

The response of the respiratory system to mental stress has been reported in [12–14], where it was shown that stress is associated with an increased respiratory rate. Vlemincx et al. also reported the effect on respiratory variability [14]; sustained nonstressful attention reduces the total respiratory variability, while mental load increases the total variability.

In this study, we will focus on the combined analysis of the cardiovascular and respiratory systems, which is motivated by the strong interaction between both systems. Respiratory sinus arrhythmia (RSA) is perhaps the best known cardiorespiratory interaction and is the phenomenon that the heart rate increases during inhalation and decreases during exhalation [15]. This influence is reflected in the HF spectral component of RRV, which is therefore often taken as a measure of RSA. Although many studies show that RSA is related to vagal control [16–18], other researches suggest that the magnitude of RSA changes with the depth of breathing (tidal volume) and the respiratory rate, independently of vagal activity [19–22]. Hence, questions arise regarding whether RSA is a true and valid measure of vagal activity. The lack of consensus about its interpretation limits its practicability.

Nonetheless, there is a strong influence of respiration on RRV, and there is a need to conduct a combined analysis during stress. This has been done in a few studies; Pattyn et al. investigated cardiorespiratory reactivity by means of RSA and separate cardiovascular and respiratory parameters and found an increase in heart rate and a decreased RSA during stress [23]. Zhang et al. reported the effects of mental tasks on cardiorespiratory synchronizations and found reduced synchronization epochs during mental arithmetic [24].

In this study we will focus on the changes in cardiorespiratory interactions during stress using time-frequency (TF) analyses. Spectral analysis has the advantage of having a clear link with physiology but has the limitation that it requires stationary signals, which is mostly not the case with physiological signals. The benefit of time domain analyses lies in the dynamic response to stress on cardiorespiratory coupling that is obtained. Time-frequency analyses combine the advantages of time and frequency domain analyses and thus can be used to analyze nonstationary signals and in particular to quantify the temporal variations of their spectral content [25, 26].

In addition, we aim to conduct partial TF analyses where influences of respiration on RRV are separated. Partial spectral analysis is a well-established, model-free, and fully data-driven technique used to modify the spectrum of a signal based on the information carried by another one [27]. A partial TF spectrum represents a modified version of the TF spectrum of signal *x*(*t*), from which the magnitudes of the components linearly related to signal *y*(*t*) have been reduced in a way which is proportional to the coherence between *x*(*t*) and *y*(*t*). This technique was previously used by Badra et al. to assess the partial coherence between RRV and systolic pressure by removing the influence that respiration exerts on both signals [28]. In this study, we will consider one partial spectrum that contains RRV related to respiration and one that is unrelated to respiration. It is important to note that we do not intend to provide a reliable estimate of vagal modulation. We intend to quantify changes in RRV power related and unrelated to respiration. The motivation to conduct this analysis originates from a previous study that showed that spectral features based on variations in the RR interval series unrelated to respiration yield an almost perfect classification (accuracy = 98%) between rest and stress, while traditional RRV analysis resulted in a classification accuracy of only 57% [29]. This result suggests that RRV unrelated to respiration contains important information about stress mechanisms.

In this paper, we will conduct (cross) TF analyses of RR interval series and respiration and assess their coherence during two similar mental stress tasks and a sustained attention task. We hypothesize finding a strong cardiorespiratory response during the first stress task and also a clear, but reduced, coupling during the second task due to habituation. The attention task is nonstressful and is hypothesized to only express a small reaction. As the interaction between RRV and respiration is vagally mediated and stress is linked to a reduced vagal control, the strength of the cardiorespiratory coupling is expected to decrease as a response to stress. The partial TF spectrum related to stress is hypothesized to mainly have HF power, while it is expected that the partial spectrum unrelated to respiration will primarily contain LF power and only little HF power.

## 2. Material and Methods

### 2.1. Experimental Setting

The database for this research consists of ECG (sampling frequency *f*_*s*_ = 1000 Hz) and respiration (*f*_*s*_ = 50 Hz) measurements of 43 healthy volunteers (age: 18–22 years) that were recorded at the Department of Psychology and Educational Sciences of the KU Leuven (Leuven, Belgium). The respiration was measured using the LifeShirt System (Vivometrics, Inc., Ventura, CA, USA), which estimates the tidal volume, further used as respiratory signal, by means of respiratory inductive plethysmography (RIP) around the ribcage and the abdomen.

During the experimental protocol, the participants were instructed to conduct two types of tasks. The first task was a nonstressful attention task where the participants had to indicate the largest number on a computer using a mouse cursor. During the second task, the students had to perform a mental arithmetic task which induces stress. The whole protocol consists of an attention task (AT) and 2 mental stress tasks (MT1 and MT2), each followed by a recovery period. The order of the tasks was randomized. Prior to any task, a resting period was recorded during which the participants watched a relaxing documentary (RD). Each task and RD had a duration of 6 minutes. For this study, RD, AT, MT1, and MT2 were used. Due to missing data, the recordings of only 40 students were included in the study.

The experiment was approved by the Ethics Committees of the Department of Psychology and Educational Sciences and of the Faculty of Medical Sciences. The study was in accordance with the Declaration of Helsinki (2008).

### 2.2. Preprocessing

The RR interval series, the signal that contains the time between two heart beats, is composed by detection of the *R* peaks in the ECG using the Pan-Tompkins algorithm [30]. All *R* peak detections are automatically verified using the algorithm described in [31] and afterwards visually inspected. Next, the respiratory signal and the RR interval series are resampled at 4 Hz using cubic spline interpolation. Both signals are high-pass filtered at 0.003 Hz to remove very slow oscillations.

All processing steps of the data are performed in MATLAB R2012a (MathWorks, Natick, MA, USA).

### 2.3. Cross Time-Frequency Analysis

The cross time-frequency spectrum *S*_*xy*_(*t*, *f*) of signals *x*(*t*) and *y*(*t*) is estimated using a time-frequency distribution (TFD) [32]:

(1) $$\begin{array}{c} S_{xy}\left(t,f\right)=\iint_{-\infty }^{+\infty }\Phi \left(\tau ,\nu \right)A_{xy}\left(\tau ,\nu \right)e^{j2\pi (t\nu -\tau f)}d\nu \;d\tau ,\\ A_{xy}\left(\tau ,\nu \right)=\int_{-\infty }^{+\infty }x\left(t+\frac{\tau }{2}\right)y^{*}\left(t-\frac{\tau }{2}\right)e^{-j2\pi \nu t}dt, \end{array}$$

where *A*_*xy*_(*τ*, *ν*) is the cross-ambiguity function. Smoothing is performed by an exponential kernel, in the ambiguity domain, defined as

(2) $$\begin{array}{c} \Phi \left(\tau ,\nu \right)=\exp\left\{-\pi {\left[{\left(\frac{\nu }{\nu_{0}}\right)}^{2}+{\left(\frac{\tau }{\tau_{0}}\right)}^{2}\right]}^{2\lambda }\right\}. \end{array}$$

In this study, values of *τ*_0_, *ν*_0_, and *λ* are set to 0.050, 0.046, and 0.3, respectively, leading to a kernel function with a TF resolution of {Δ_*t*_, Δ_*f*_} = {10.9 s, 0.039 Hz}, where Δ_*t*_ and Δ_*f*_ quantify the spreading introduced by the kernel [25, 32].

Time-frequency coherence is an estimate of the strength of the local coupling between two signals and is determined by

(3) $$\begin{array}{c} \gamma_{xy}\left(t,f\right)=\frac{\left|S_{xy}\left(t,f\right)\right|}{\sqrt{S_{xx}\left(t,f\right)S_{yy}\left(t,f\right)}},\;\gamma_{xy}\left(t,f\right)\in \left[0,1\right]. \end{array}$$

Time-frequency phase difference (TFPD) is given by

(4) Θxy(t,f)=arctan[ℑ[Sxy(t,f)]ℜ[Sxy(t,f)]], Θxy(t,f)∈[−π,π].

### 2.4. Partial Time-Frequency Analysis

The separation of respiratory influences from the RR interval series is conducted using partial TF spectra obtained by

(5) Sxx/y(t,f)=Sxx(t,f)−Sxy(t,f)Syx(t,f)Syy(t,f)=(1−γxy2(t,f))Sxx(t,f).

In this study, we focus on the partial spectrum of the RRV (*x* = *R*) from which the respiratory influences (*y* = *r*) are removed (*S*_RR/*r*_(*t*, *f*)). Its complement, that is, the RRV which contains RR changes related to respiration (*S*_RR,*r*_(*t*, *f*)), is defined as

(6) $$\begin{array}{c} S_{xx,y}\left(t,f\right)=\gamma_{xy}^{2}\left(t,f\right)S_{xx}\left(t,f\right). \end{array}$$

**Figure 1:**
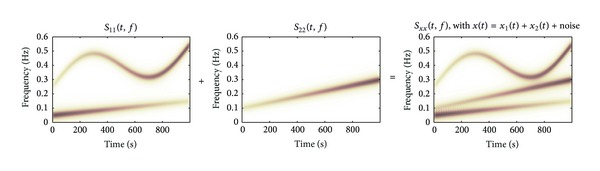
Time-frequency spectra of signals *x*_1_(*t*), *x*_2_(*t*), and *x*(*t*), see (7)-(8), of simulation study 1. Note that *x*_1_(*t*) and *x*_2_(*t*) do not share a spectral component.

**Figure 2:**
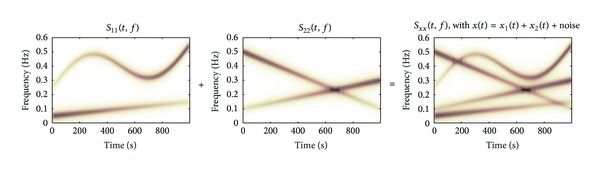
Time-frequency spectra of signals *x*_1_(*t*), *x*_2_(*t*), and *x*(*t*), see (7)-(8), of simulation study 2. Note that spectral components of *x*_1_(*t*) and *x*_2_(*t*) intersect in the TF domain.

In order to evaluate the performance of partial TF spectra, a simulation study is set up. Let *x*_1_(*t*) and *x*_2_(*t*) be two nonstationary signals, with each one being composed by one or two different complex exponentials showing both amplitude (*A*_*k*_(*t*)) and frequency (*f*_*k*_(*t*)) modulations:

(7) $$\begin{array}{c} x_{i}\left(t\right)=\sum_{k=1}^{M_{i}}A_{k}\left(t\right)\exp\left(j\left(\phi_{k}\left(t\right)+\theta_{k}\right)\right) \end{array}$$

with *f*_*k*_(*t*) = (1/2*π*)(*dϕ*_*k*_(*t*)/*dt*), *i* = {1,2}, and *M*_*i*_ is the number of spectral components. Let *x*(*t*) be given as,

(8) $$\begin{array}{c} x\left(t\right)=x_{1}\left(t\right)+x_{2}\left(t\right)+\xi \left(t\right), \end{array}$$

where *ξ*(*t*) is complex zero-mean white Gaussian noise whose standard deviation is adjusted to obtain an SNR equal to 20 dB. In a first simulation study, *M*_1_ and *M*_2_ are, respectively, 2 and 1. The TF spectra *S*_11_(*t*, *f*) and *S*_22_(*t*, *f*) are given in Figure 1. In a second simulation study, an extra component is added to *x*_2_(*t*), yielding signals with TF spectra shown in Figure 2.

In the next step of the simulation study, the partial spectrum *S*_*xx*/2_(*t*, *f*) is computed. The performance of partial TF spectra is evaluated based on the agreement between *S*_*xx*/2_(*t*, *f*) and *S*_11_(*t*, *f*). This agreement is quantified by the spectral distance *d* between both TF spectra [33]:

(9) $$\begin{array}{c} d=\frac{{||\bar{S_{xx/2}}\left(t,f\right)-S_{11}\left(t,f\right)||}_{l_{1}}}{{||S_{11}\left(t,f\right)||}_{l_{1}}} \end{array}$$

with $\bar{S_{xx/2}}(t,f)$ being the mean partial TF spectrum estimated over all of the realizations of each simulation study and ||*A*||_*l*_1__ being the L1-norm of matrix *A*.

### 2.5. Derivation of Time-Varying Parameters

The time courses of several indices that characterize the interactions between RRV and respiration are derived. We compute the following time-varying parameters for TF spectrum *S*_*α*_(*t*, *f*)  ∈[*S*_RR_(*t*, *f*), *S*_RR/*r*_(*t*, *f*), *S*_RR,*r*_(*t*, *f*)] in specific frequency bands *β*, with *f*_*β*_ being the frequencies in *β*.

- Instantaneous power is given as *P*_*α*_^*β*^(*t*) = ∑_*β*_ *S*_*α*_(*t*, *f*_*β*_)*δ*_*f*_ with *δ*_*f*_ being the frequency step in the spectrum.
- Instantaneous frequency *F*_*α*_^*β*^(*t*) is estimated as the frequency of the spectral peak in frequency band *β*.

The considered bands *β* are based on the traditional RRV frequency bands:

- *β*_LF_ = [0.04 Hz, 0.15 Hz],
- *β*_HF_ = [0.15 Hz, 0.40 Hz],
- *β*_TOT_ = [0.04 Hz, 0.40 Hz].

In addition, two parameters that describe the local coupling between RRV and respiration in a specific frequency band *β*_*r*_ around respiration are computed as follows:

- coherence *γ*_*Rr*_^*β*_*r*_^(*t*) = mean_*f*∈*β*_*r*__[*γ*_*Rr*_(*t*, *f*)],
- phase difference Θ_*Rr*_^*β*_*r*_^(*t*) = mean_*f*∈*β*_*r*__[Θ_*Rr*_(*t*, *f*)],

where *β*_*r*_(*t*) is a time-varying band defined as *F*_*r*_(*t*)±(Δ_*f*_/2) with *F*_*r*_(*t*), the respiratory frequency. This frequency band is centered around the breathing frequency and lies generally within the traditional HF band.

The last two time-varying parameters that are considered are the instantaneous respiratory frequency, *F*_*r*_(*t*), and heart rate, HR(*t*), expressed in beats per minute [bpm].

### 2.6. Statistical Analysis

The Wilcoxon signed rank test is used to assess statistical differences between the 4 conditions (AT, MT1, MT2, and RD). Because we are interested in the dynamic response to each task, statistical analysis was conducted sample by sample to track the *P* values in time. Statistical significance is obtained when *P* < 0.05.

In the case of the instantaneous frequency and power, we are only interested in the relative changes, regardless of the subject's general condition or prior influences. Therefore, a correction was applied at the onset of each task prior to application of the Wilcoxon signed rank test to study only the relative response. The reference is taken as the mean value of each parameter in a window Δ_*t*_ around the onset of each task. No correction is applied for the coherence and phase difference.

## 3. Results

### 3.1. Simulation Study

**Figure 3:**
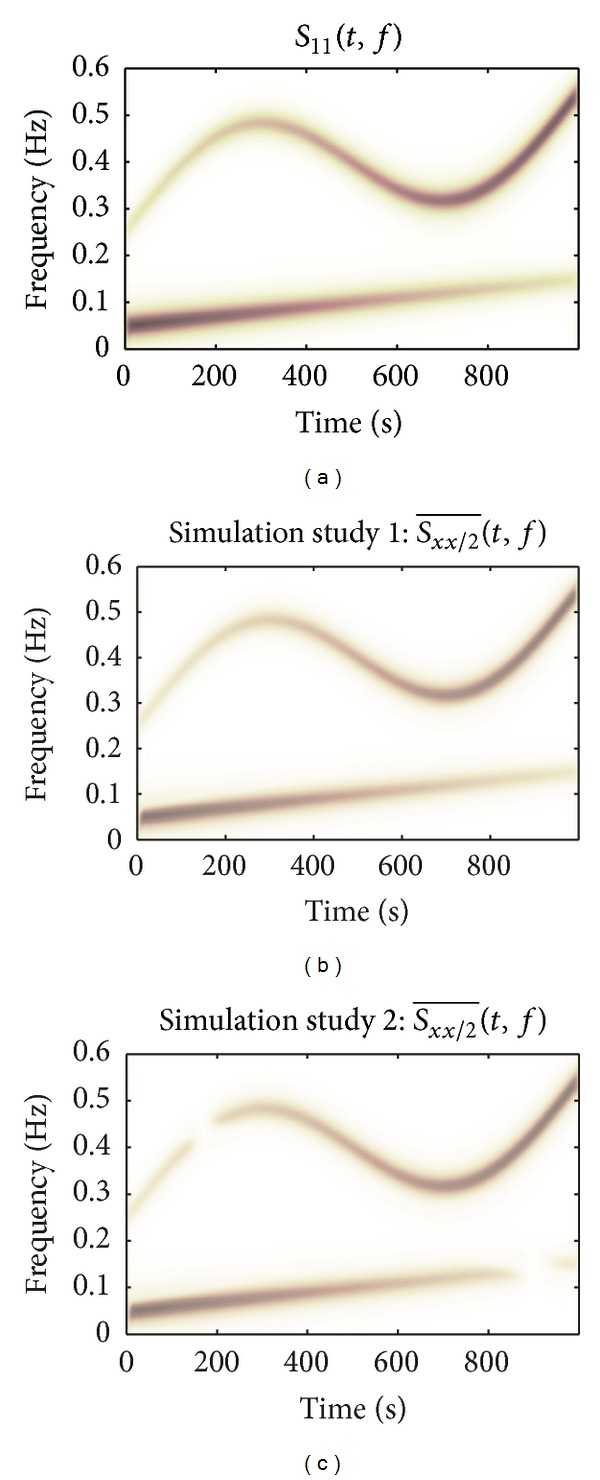
Averaged results of the simulation study: (a) TF spectrum *S*_11_(*t*, *f*); (b) partial TF spectrum $\bar{S_{xx/2}}(t,f)$ of simulation study 1; (c) partial TF spectrum $\bar{S_{xx/2}}(t,f)$ of simulation study 2. In the ideal case, the partial spectrum $\bar{S_{xx/2}}(t,f)$ equals *S*_11_(*t*, *f*).

Each simulation study is performed using 50 different realizations of noise and phases *θ*_*k*_, derived from a uniform random distribution between −*π* and *π*. The presented results arise from averaging over these realizations.  Figure 3 shows the averaged results of the simulation studies described in Section 2.4. In Figure 3(a), the TF spectrum *S*_11_(*t*, *f*) is given.  Figure 3(b) displays the partial TF spectrum $\bar{S_{xx/2}}(t,f)$ of the first simulation. A high similarity (*d* = 0.20) between $\bar{S_{xx/2}}(t,f)$ and *S*_11_(*t*, *f*) is found in the first simulation.

**Figure 4:**
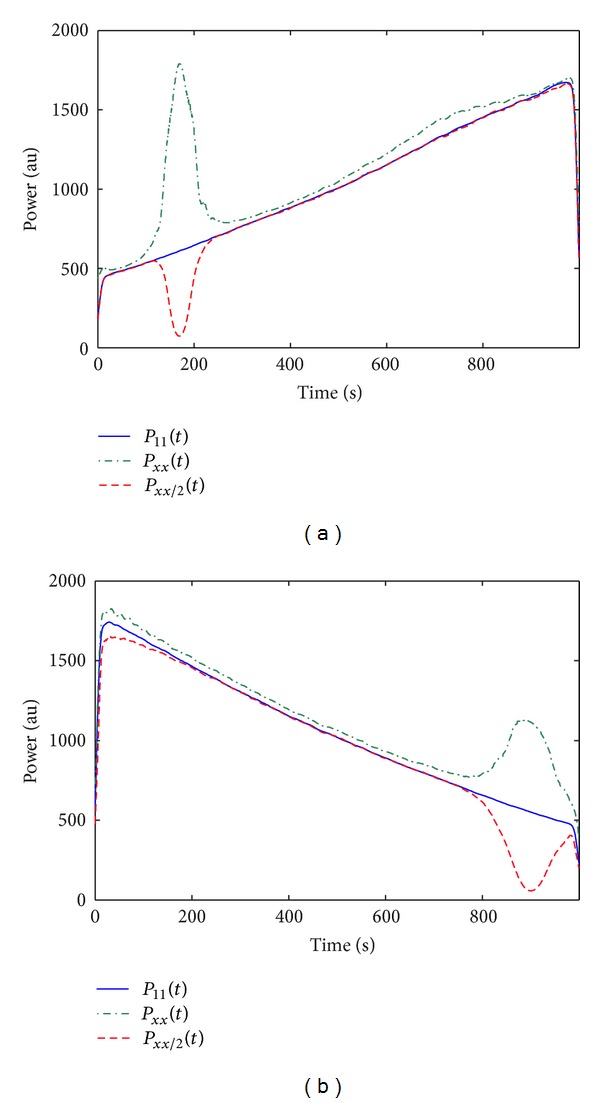
Averaged instantaneous power of the two spectral components of *x*_1_(*t*) in simulation study 2 of *S*_11_(*t*, *f*), *S*_*xx*_(*t*, *f*), and *S*_*xx*/2_(*t*, *f*). (a) component 1 of *x*_1_(*t*), which corresponds to the sinusoidal component; (b) component 2 of *x*_1_(*t*), which corresponds to the linear component.

The result of the second simulation study is shown in Figure 3(c). We can observe “missing” parts around *t* = 170 s and *t* = 900 s, precisely for those portions of the TF domain in which spectral components of *x*_1_(*t*) and *x*_2_(*t*) intersect. A higher spectral distance *d* = 0.22 is found, indicating a slightly lower agreement between $\bar{S_{xx/2}}\left(t,f\right)$, and *S*_11_(*t*, *f*).  Figure 4 shows the instantaneous power of the spectral components of *x*_1_(*t*) in simulation study 2 of *S*_11_(*t*, *f*), *S*_*xx*_(*t*, *f*) and *S*_*xx*/2_(*t*, *f*), computed as the mean power in a window Δ_*f*_ around the frequency modulation *f*_*k*_(*t*) from (7). Components 1 and 2 correspond, respectively, to the sinusoidal and linear components of *x*_1_(*t*) with a linear increasing and decreasing power (see *P*_11_(*t*)). Remark that the instantaneous power of *S*_*xx*_(*t*, *f*) significantly increased around *t* = 170 s and *t* = 900 s, precisely when the instantaneous frequencies of *x*_1_(*t*) and *x*_2_(*t*) intersect. The instantaneous power of *S*_*xx*/2_(*t*, *f*) decreased in correspondence to these intersections.

### 3.2. Stress Monitoring

**Figure 5:**
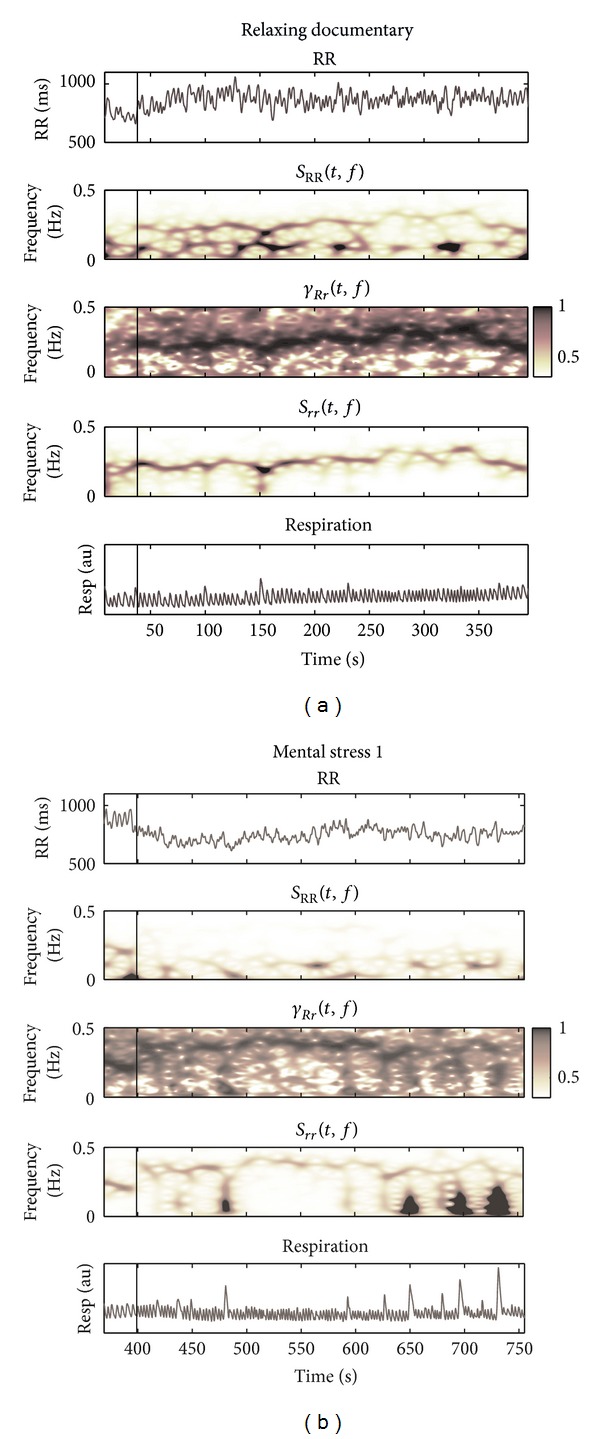
Example of RR interval series (RR) and respiratory (resp) signal and their TF spectra (*S*_RR_(*t*, *f*), *S*_*rr*_(*t*, *f*)) and coherence (*γ*_*Rr*_(*t*, *f*)) during documentary watching and the first mental stress task. Vertical lines indicate the onset of RD (a) and MT1 (b).

Figure 5 shows the RR interval series, respiratory signal, and TF spectra and coherence of one typical subject during the documentary watching (RD) and the first mental stress task. In the top 2 panels it is shown that both RR and RRV decrease when MT1 is compared with RD for this subject. An increase in the respiratory rate and an increased number of sighs are noted during mental stress. A high coherence around respiratory frequency is found during RD, while a reduction in coherence is noticed during stress for this subject.

**Figure 6:**
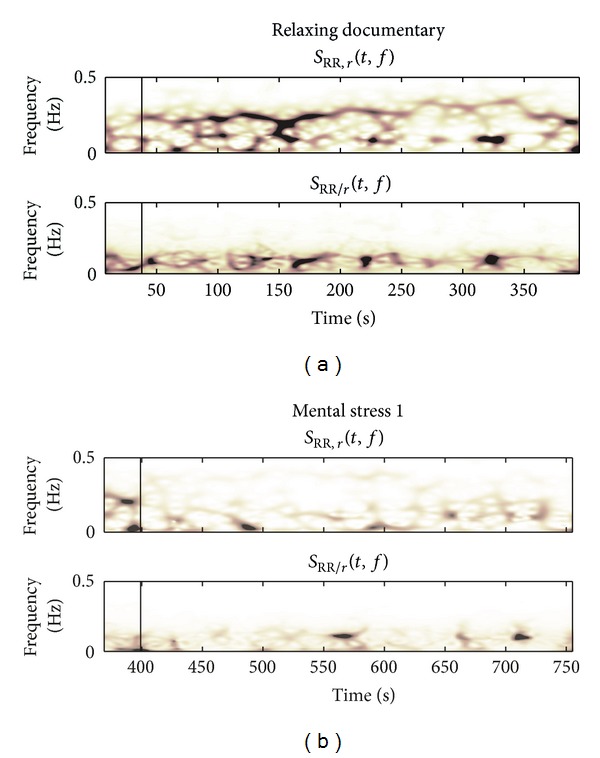
Example of partial TF spectra (*S*_RR,*r*_(*t*, *f*), *S*_RR/*r*_(*t*, *f*)) during documentary watching and the first mental stress task. Vertical lines indicate the onset of RD (a) and MT1 (b).

Figure 6 shows the partial TF spectra for the same subject during RD and MT1. During RD, we observe that *S*_RR,*r*_(*t*, *f*) contains power in both the LF and HF bands. On the other hand, *S*_RR/*r*_(*t*, *f*) includes most power in the LF band. However, note that the HF band still comprises some power, though strongly reduced. *P*_RR,*r*_(*t*) and *P*_RR/*r*_(*t*) decreased during MT1 in comparison to RD.

#### 3.2.1. Time-Varying Parameters

**Figure 7:**
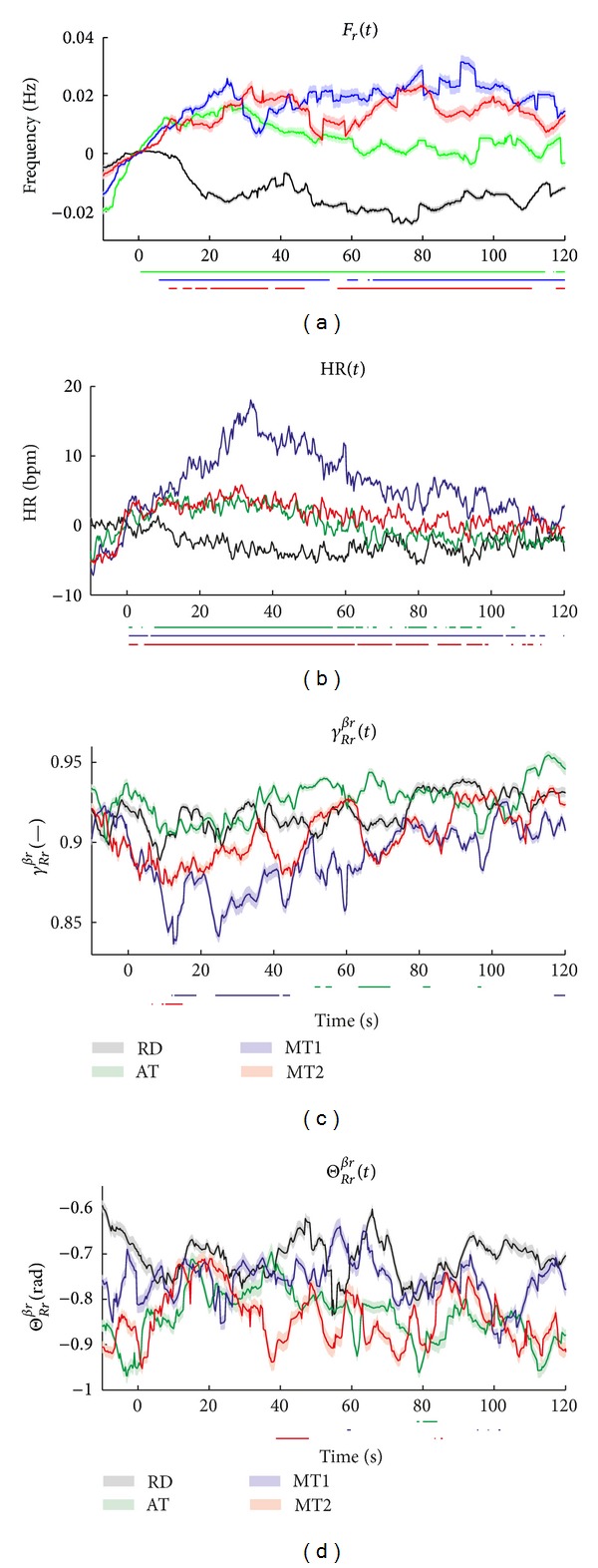
Median instantaneous respiratory frequency (*F*_*r*_(*t*)), heart rate (HR(*t*)), coherence (*γ*_*Rr*_^*βr*^(*t*)), and phase difference (Θ_*Rr*_^*βr*^(*t*)) in the time-varying band *β*_*r*_(*t*). The standard error is shaded. The bars below each subplot indicate the time instances of significant differences between RD and the tasks.

Figure 7 shows the median instantaneous respiratory frequency (*F*_*r*_(*t*)) and heart rate (HR(*t*)) in the top panels. In addition, the time instances of statistically significant differences between RD and the other tasks are indicated by bars below each subplot. Both *F*_*r*_(*t*) and HR(*t*) increase during AT, MT1, and MT2, while they slightly decrease during RD. Significant differences between RD and the other conditions are observed within 10 s after onset of each task, throughout the whole task for *F*_*r*_(*t*). No differences are found between AT, MT1, and MT2. In the heart rate, significant differences are found a few seconds after onset of each task, between all conditions, except between AT and MT2. The heart rates differ maximally after 30 s from the onset of the task. After 100 s, no consistent differences between the 4 conditions are observed.

The coherence (*γ*_*Rr*_^*β*_*r*_^(*t*)) and phase difference (Θ_*Rr*_^*β*_*r*_^(*t*)) are shown in the lower panels of Figure 7. Mental stress exhibits a reduction in cardiorespiratory coherence compared to RD and AT. This is only significant for MT1. No other differences in coherence between all conditions can be observed. The phase difference does not show any consistent difference as a result of mental stress or attention.

**Figure 8:**
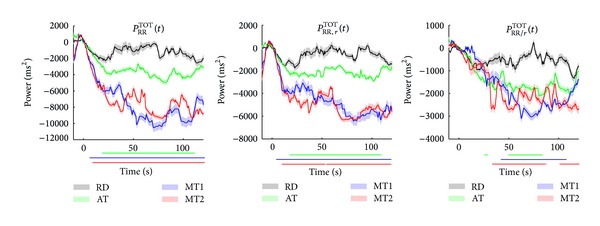
Median instantaneous power for TF spectra *S*_RR_(*t*, *f*), *S*_RR,*r*_(*t*, *f*), and *S*_RR/*r*_(*t*, *f*) in the total frequency band. The standard error is shaded. The bars below each subplot indicate the time instances of significant differences between RD and the tasks.

Figure 8 shows the median instantaneous power in the total frequency band. *P*_RR_^TOT^(*t*) decreases during mental stress. This reduction is statistically significant already 10 s after onset of the mental task. Also a significant, but smaller, reduction is found during AT after 20 s. When considering the partial spectra, we observe a similar pattern in *P*_RR,*r*_^TOT^(*t*). *P*_RR/*r*_^TOT^(*t*) shows statistically significant differences between RD and MT1 from 40 s till 100 s, between RD and MT2 from 30 s till 120 s, and between RD and AT from 45 s till 80 s.

**Figure 9:**
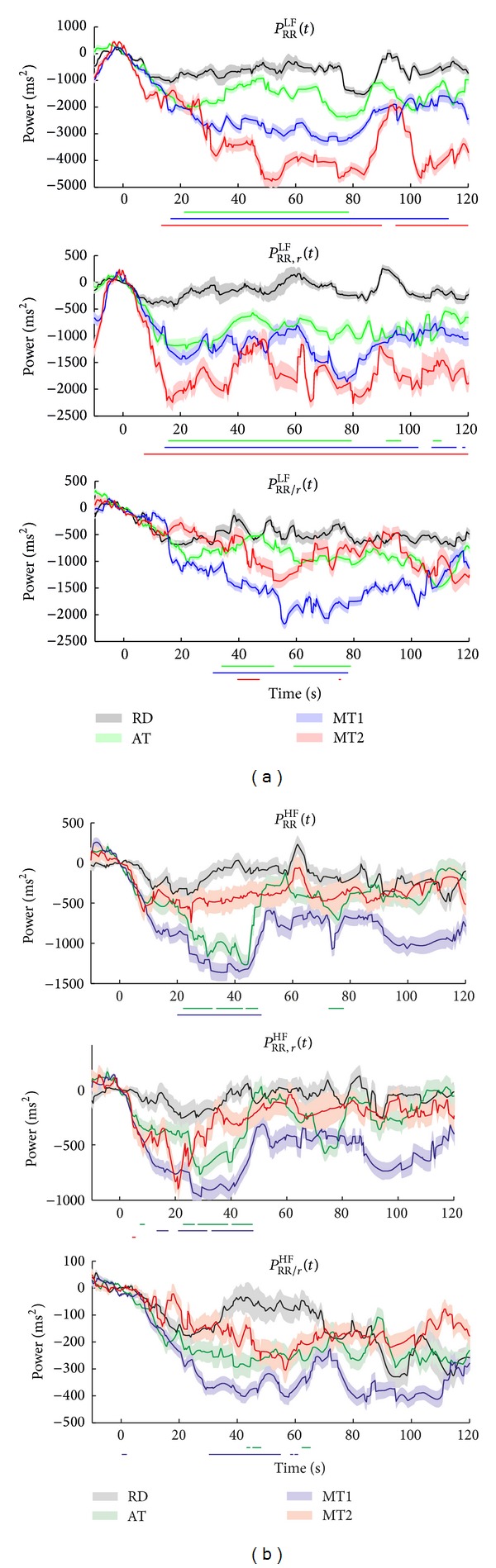
Median instantaneous power for TF spectra *S*_RR_(*t*, *f*), *S*_RR,*r*_(*t*, *f*), and *S*_RR/*r*_(*t*, *f*) in the LF (a) and HF (b) bands. The standard error is shaded. The bars below each subplot indicate the time instances of significant differences between RD and the tasks.

Figure 9 shows the median instantaneous power for TF spectra *S*_RR_(*t*, *f*), *S*_RR,*r*_(*t*, *f*), and *S*_RR/*r*_(*t*, *f*) in the LF, Figure 9(a), and HF, Figure 9(b), bands. Interestingly, *P*_RR_^LF^(*t*) displays a significant reduction during MT2. This reduction is also found during MT1 and AT compared to RD, but to a lesser extent. A similar pattern is observed in *P*_RR,*r*_^LF^(*t*), suggesting that this reduction may be related to respiration; *P*_RR/*r*_^LF^(*t*) shows only a significant reduction during MT1 and AT, but not during MT2.

In the HF band of *S*_RR_(*t*, *f*), we observe significant differences between 20 s and 50 s when comparing MT1 and AT with RD. This reduction is also found in *P*_RR,*r*_^HF^(*t*) and to a lesser, but still significant, extent in *P*_RR/*r*_^HF^(*t*) (only for MT1), showing that the differences that appear in the HF band can be attributed to both RRV related and unrelated to respiration. In contrast with the findings in the LF band, MT2 shows no significant difference with any of the other conditions.

**Figure 10:**
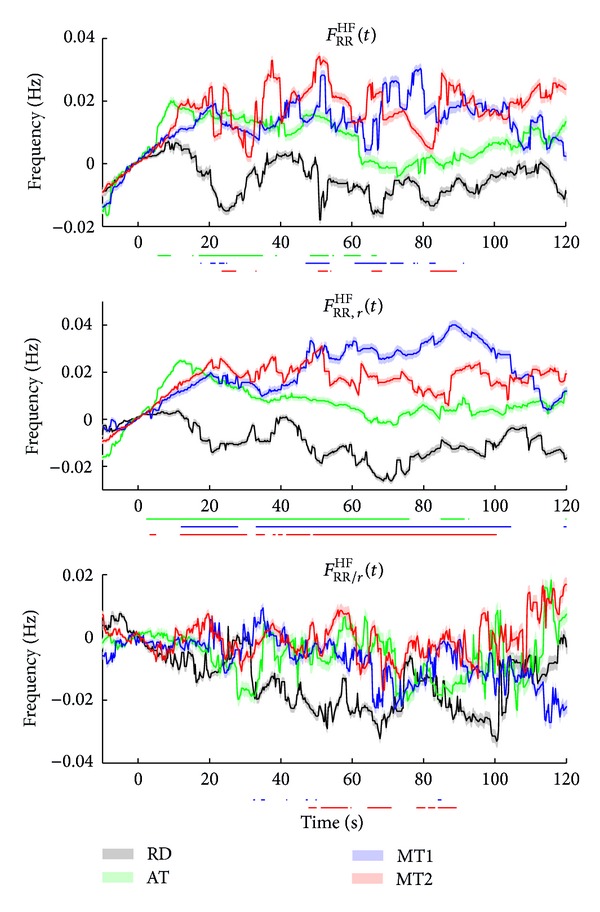
Median instantaneous frequency for TF spectra *S*_RR_(*t*, *f*), *S*_RR,*r*_(*t*, *f*), and *S*_RR/*r*_(*t*, *f*) in the HF band. The standard error is shaded. The bars below each subplot indicate the time instances of significant differences between RD and the tasks.

The median instantaneous frequencies for TF spectra *S*_RR_(*t*, *f*), *S*_RR,*r*_(*t*, *f*), and *S*_RR/*r*_(*t*, *f*) in the HF band are given in Figure 10. The instantaneous frequencies in the LF band are not shown as they do not exhibit significant differences between the conditions. The top figure shows *F*_RR_^HF^(*t*), which is expected to be highly coupled to respiration. This is confirmed when looking at *F*_RR,*r*_^HF^(*t*), which shows a highly similar pattern as *F*_*r*_(*t*) in Figure 7. Also here, significant differences between AT, MT1, and MT2 and RD are found. *F*_RR/*r*_^HF^(*t*) shows no distinction between the tasks.

## 4. Discussion

In this work, we aimed to characterize stress-related changes in cardiorespiratory interactions. We used time-frequency analyses to assess the cardiorespiratory coupling. In addition, partial TF spectra were introduced to distinguish between RR interval variations related and unrelated to respiration. This approach was also evaluated in two simulation studies. Next, several time-varying parameters were derived from the computed (partial) TF spectra and statistical analysis was conducted to assess whether different mental tasks provoked different cardiorespiratory responses compared to changes induced by a relaxing documentary watching task.

### 4.1. Cross and Partial Time-Frequency Analyses

Time-frequency analyses were conducted as they provide the time course of spectral indices which have an established physiological interpretation. The proposed TFD was used in previous research [25, 26, 32] and proved to provide a better TF resolution and localization than spectrogram and continuous wavelet transform of the local coupling between two signals.

The use of partial TF analyses, as described in Section 2.4, was evaluated in two simulation studies, where the performance was assessed by the similarity between *S*_11_(*t*, *f*) and $\bar{S_{xx/2}}(t,f)$. In simulation study 1, none of the signal spectral components intersect; that is, they have no overlapping instantaneous frequencies. As shown in Figures 3(a) and 3(b), there is a high correspondence between *S*_11_(*t*, *f*) and $\bar{S_{xx/2}}(t,f)$, which demonstrates that a good performance is obtained in the case that two signals do not share a spectral component.

In simulation study 2, the instantaneous frequencies of *x*_1_(*t*) and *x*_2_(*t*) overlap twice, around *t* = 170 s and *t* = 900 s, where *P*_*xx*_(*t*) increases and $\bar{S_{xx/2}}(t,f)$ is lower than *S*_11_(*t*, *f*). A strong reduction in $\bar{S_{xx/2}}(t,f)$ is expected because in these portions of the TF domain, *γ*_*xy*_^2^(*t*, *f*) ~ 1, by definition, partialization is achieved by removing *γ*_*xy*_^2^(*t*, *f*)*S*_*xx*_(*t*, *f*) from *S*_*xx*_(*t*, *f*). From this second simulation study, we can conclude that partial TF spectra cannot be used to separate the contribution of two spectral components which are simultaneously oscillating at the same instantaneous frequency. However, this limitation is expected to have little impact on the analysis of cardiorespiratory interactions for the following reasons: (1) RR oscillations locally coupled to and synchronous with respiration are assumed to come from respiration itself; (2) the hypothetical overlapping between RRV oscillations related to and not related to respiration is expected to be low, because the respiratory signal is relatively narrow banded; and (3) as long as the difference between the instantaneous frequencies of the spectral components of different signals is lower than the frequency resolution (Δ_*f*_), the partial spectrum correctly separates them. A critical evaluation on partial spectral analysis can be found in [34].

In addition, (5) shows that the amount of power in the partial TF spectrum *S*_*xx*/*y*_(*t*, *f*) which is removed from *x*(*t*) around (*t*_0_, *f*_0_) is proportional to the coherence between *x*(*t*) and *y*(*t*) around (*t*_0_, *f*_0_). If at time *t*_0_ *x*(*t*) and *y*(*t*) share a spectral component with the same instantaneous frequency *f*_0_, their coherence at (*t*_0_, *f*_0_) will be close to 1 and thus the component will be removed, regardless of the amplitude modulation of both components.

Finally, it is important to note that the use of partial TF spectra is motivated by the fact that this approach is (1) nonparametric; that is, it does not rely on any model, and (2) it is fully data driven, in contrast to other techniques such as TF filtering: (1) model-based approaches, such as the IPFM model, MVAR models, or multivariate point process models, offer the opportunity to estimate hidden variables and the strength of directional couplings, but their outcomes strongly depend on the goodness-of-fit of the model. For this reason, we opted for a methodology which, in our view, is more robust to evaluate changes in RRV linearly related and unrelated to respiration. (2) TF filtering requires the use of a TF mask or a smoothing function, where it is necessary to decide on the geometry of the mask, its values, and, more importantly, its functioning. This makes the algorithm specific to a given set of signals in a given condition. Our approach, on the contrary, being data driven, can be generalized to any kind of signal in any possible situation and provides an easier interpretation of the results as the magnitudes of the partial spectra are proportional to the coherence between RRV and respiration.

### 4.2. Cardiorespiratory Response

The heart rate shows a clear increase due to mental stress and sustained attention. These findings are in agreement with those reported in [6, 9, 10, 23]. In line with the hypothesis, the largest increase is found during MT1, while a smaller increase is observed during MT2 and AT, suggesting a positive correlation between HR and mental load.

Also in line with our hypothesis is the decrease in the total power of RRV that is more pronounced during the mental tasks than during the attention task. Watching the documentary also gives rise to a brief reduction in total power immediately after onset of the task. These results suggest that mental stress causes vagal withdrawal, and sustained attention also a small reduction in vagal activity.

As previously reported in [9], the strongest reduction of HF power corresponds to MT1. Moreover, this study reveals that the reduction during AT is mainly due to the initial drop in HF between 20 and 50 s. The effect of MT1 is also strongest during this interval, but a small reduction persists throughout the whole task. In contrast to what was found in [9], no significant difference between MT2 and RD was observed, indicating that habituation might have occurred during MT2. The influence of the second mental stress task is only seen in the LF band, which corresponds to both sympathetic and vagal influences. A small reduction is found during AT and MT1. These results are in contrast with the findings by Taelman et al. using the original RR interval series [9]. They found a strong reduction in LF power during MT1 and AT, but MT2 did not differ significantly from RD. When conducting TF analyses, they did report significant differences between AT and MT2, but no differences between AT and MT1. We can observe a similar pattern; that is, the largest difference is between AT and MT2. However, these differences are not significant in this study. The differing results might arise from the applied correction to study only relative changes, thereby discarding possible effects from prior tasks. This was not implemented in [9].

The respiratory frequency increases during AT and MT, which was also reported in [14]. This is an important parameter to take into account as many authors suggest that an inverse relationship between RSA magnitude and respiratory rate exists, which is independent of vagal activity [19–22]. Seeing that HF power is often taken as a measure of RSA, it is of utmost importance to be able to distinguish changes in HF due to vagal control or due to changes in respiratory rate. The same reasoning holds for the depth of breathing, as it is also shown that this influences RSA magnitude independently of vagal outflow. In this TF framework, information on the depth of breathing can be obtained in *P*_*rr*_^*β*_*r*_^(*t*). We found, however, that, in this study, the depth of breathing did not differ significantly among the tasks or compared to RD.

We observed that the coherence slightly decreases during stress. This effect as a result to mental stress was hypothesized, due to vagal withdrawal and sympathetic activation [17]. The phase difference remains constant during the different conditions and is thus not affected by stress or attention.

#### 4.2.1. Partial TF Analysis

Figure 9 shows that there are similar time courses in the 4 conditions between *P*_RR_(*t*) and *P*_RR,*r*_(*t*) in the LF and HF bands. This indicates that both the RR interval variations in the LF and HF bands are highly coupled to respiration and demonstrates the dominant effect of respiration on RRV, as can also be observed in the instantaneous high frequency in Figure 10. However, the question rises regarding what the physiological interpretation of this cardiorespiratory coupling in the LF band is. Note that during the computation of indices from the LF band we verified that the respiratory frequency did not fall within this band. If we did encounter slow respiratory rates, we did not compute the indices at those time instances. Possibly, the respiratory influences in the LF band can be explained by the baroreflex feedback theory or irregular breathing patterns, as was suggested by Yildiz and Ider [35].

Also in the total band, the effect of respiration is apparent. Moreover, the effects of stress in *P*_RR,*r*_^TOT^(*t*) are already pronounced after 3–7 s, while in *P*_RR_^TOT^(*t*) a significant difference was only found after 10 s. Both in *P*_RR_^TOT^(*t*) and *P*_RR,*r*_^TOT^(*t*) the effect of sustained attention is detected after 20 s.

As expected, most of the power in the HF band is related to respiration. However, there is still some HF power in *S*_RR/*r*_(*t*, *f*), which furthermore shows effects related to stress. Opposed to what we hypothesized, *P*_RR/*r*_^LF^(*t*) does not show a clear discrimination between the conditions. Only a slight reduction is found during MT1 and AT. The analysis of *P*_RR/*r*_^TOT^(*t*) exhibits differences between RD and the other tasks; the effects of sustained attention appear only for a short interval, from 45 to 80 s, while the influence of stress on physiological processes, other than respiration, is observed after 30 s and lasts till 100 s.

The results from partial TF analysis suggest that the effects of stress are mainly related to respiration. Although it was hypothesized that RRV unrelated to respiration would show enhanced stress influences, as was found during classification of rest and stress [29], we found only small differences between the active tasks and RD. The partial TF analyses revealed different temporal patterns as a result of stress; a fast response (within 7 s) is observed in RRV related to respiration, as shown in Figure 8, while RR interval variations unrelated to respiration are only apparent after 30 s.

Finally, it is important to note that the results not only display different responses to mental stress and attention, but also differences between MT1 and MT2 can be observed. Although both tasks are the same, they present a different response, in terms of magnitude and latency. We hypothesized that the response to stress would be faster and stronger during MT1, while during MT2 the effect of stress would be reduced due to habituation, as was also observed by Taelman et al. [9]. This hypothesis is confirmed in terms of heart rate, coherence, HF, and total power.

## 5. Conclusion

The goal of this study was to characterize the dynamic interactions in the cardiorespiratory regulation in response to mental stress. As a suitable approach, cross time-frequency analyses were conducted. In addition, partial TF spectra were computed to evaluate separately the response of RR interval variations linearly related to respiration and variations that are not linked to respiration. Although stress also influences the respiratory pattern [14], breathing is also under voluntary control and might not always be a suitable indicator of stress.

Sustained attention was adopted as a nonstressful task during which we observed an increased heart and respiratory rate and a slightly increased coherence. A transitory reduction of HF power suggests a vagal withdrawal during the first minute. Partial TF analyses showed that the response to sustained attention of RR interval variations related to respiration differs from that to documentary watching after 20 s, while RRV unrelated to respiration exhibits a significant response after 45 s. After 80 s, there is no effect detected of this attention task.

We found that mental stress causes an increase in heart rate and respiratory rate. Also a reduction in cardiorespiratory coherence, HF power, and LF power was found, indicating vagal withdrawal. The partial TF analysis revealed that the response to stress of RR interval variations related to respiration appears very fast (3 s), while the variations unrelated to respiration react with a slower temporal pattern (30 s). These results demonstrate that cross and partial time-frequency analyses carry valuable information on the cardiorespiratory stress mechanisms and suggest that this is a useful tool for biofeedback in stress-reducing therapies.
